# Reevaluating Value-Based Care in Telemedicine: Clinical Opportunities in the Postpandemic Era

**DOI:** 10.1089/tmj.2024.0523

**Published:** 2024-12-06

**Authors:** Denise Soltow Hershey, David Buzanoski, Supratik Rayamajhi, Drew Murray

**Affiliations:** ^1^ Michigan State University College of Nursing, East Lansing, Michigan, USA.; ^2^ Michigan State University College of Human Medicine, East Lansing, Michigan, USA.; ^3^ Michigan Health Endowment Fund, East Lansing, Michigan, USA.

**Keywords:** telehealth, telemedicine, value-based care, pandemic

## Abstract

Telehealth during the COVID-19 pandemic became one of the main means for patients to access the health care system. Rules, regulations, and reimbursement policies were loosened, allowing for its expansion into the clinical arena. Since the end of the pandemic, virtual care models have expanded. With a larger emphasis on value-based care, there is a need to understand how telehealth can be utilized to increase value, improve access, enhance the patient experience, improve outcomes, and decrease health inequalities. The article explores the use of telehealth as it relates to a value-based care model, which includes the patient experience, quality of care (access and health equity), provider/clinical practice, and health system/financial. Recommendations for strengthening the use of telehealth to ensure value-based care are provided.

## Introduction

Virtual care models, asynchronous patient encounters, and leveraging electronic medical records (EMR) capabilities to improve panel management and population-based health have become established and trusted tools that are revolutionizing how we define “value” in health care. The COVID-19 pandemic provided an impetus for the rapid acquisition and implementation of new tools geared toward changing the way in which health care is delivered. American health care organizations, payors, providers, and patients have embraced aspects of these newly implemented technologies and have incorporated them into mainstream care. This explosion in novel health care delivery methods, occurring particularly over the past 4 years, has become the expectation of patient care, not the exception. However, implementation and adoption of these technologies remain highly variable between practices, organizations, and payors. Additionally, implementation of these principles has oftentimes worsened disparities based on social determinants of health, further reducing health equity, not improving it. As the Center for Medicare and Medicaid Services (CMS) continues to emphasize a move toward value-based care, ongoing discussions need to better define how “value” should be defined as it pertains to clinical practice and opportunities, including the use of telehealth.

## What is Telehealth?

Forms of telemedicine have been around for about 100 years. Telehealth as we know it today emerged from the fundamental framework of telemedicine.^1^ Telehealth is considered the use of some form of technology to exchange health information from one site to another. This can be synchronous (use of audio and/or video technologies) or asynchronous (such as the use of remote monitoring devices or messaging through patient portals). The CMS defines telehealth as a two-way, real-time interactive communication between a patient and the physician or practitioner at a distance site through telecommunication equipment that includes, at a minimum, audio and visual equipment.^2^

The benefits of telehealth are well known, which include (1) most elements of care can be provided, such as preventative and ongoing care, education, and counseling; (2) exposure to infectious diseases is reduced for both patients and providers; (3) the ability for patients to receive timely care; and (4) increased access to care and potentially decreased health inequities.^1,3^ Barriers to the use and implementation of telehealth have also been reported, which include (1) patient preference, (2) perception from patients that services are not equivalent between in-person and telehealth visits, (3) economic disparities such as access to computers and other equipment, (4) availability of adequate internet/broadband services, and (5) access and continuity of care that payors, licensor, HIPAA, and provider preferences may influence.^1,3,4^ To work toward overcoming these barriers, we need to understand the relationship between value-based care and telehealth, and how telehealth can potentially increase value for providers and patients.

## Value-Based Care and Telehealth

**Fig. 1.**
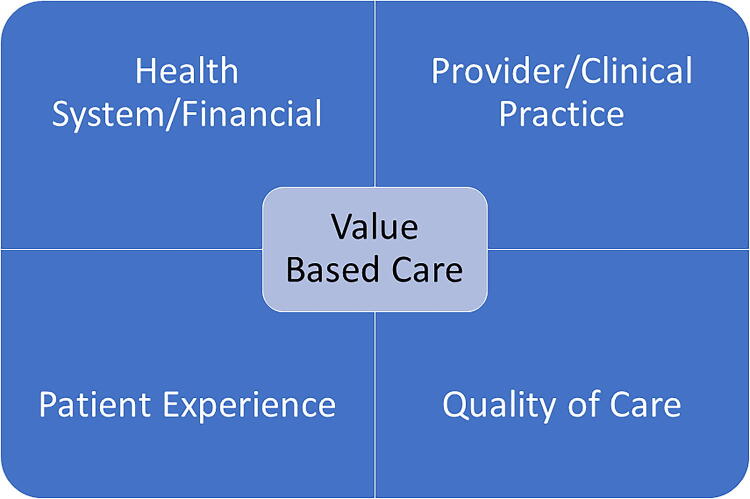
Proposed value-based care model for telehealth.

Value-based care emphasizes on quality of care, which includes access to and equity of health care, provider performance, and the patient’s experience. However, there is no doubt that affordability and financial efficiency remain central to providing health care and are an unspoken component related to the delivery of value-based care. *Figure 1* depicts these four components of value-based care, and each will be discussed further.

### HEALTH SYSTEM/FINANCIAL

When considering how to advance telemedicine in the postpandemic era, introspection and review of cost-effectiveness and financial benefits to clinical practices will be central to any next steps. Transparent publication of how the implementation of telemedicine reduces health care delivery costs needs to become the standard. During the COVID-19 pandemic reimbursement policies for telehealth services, including the ability for patients to receive services in their homes and allowing the use of technological devices such as smartphone apps.^5^ Currently, reimbursement for telehealth services is being reevaluated. The use of telehealth has been shown to improve access to and equity of care. However, it remains unknown if the use of telehealth, along with the cost of implementation and equipment, will continue to be supported by reimbursement from payors. Evidence supports that appropriate reimbursement can make telehealth services cost-effective particularly with regard to managing chronic conditions such as type two diabetes and heart failure.^5^

### PROVIDER/CLINICAL PRACTICE

During the COVID-19 pandemic, the use of telehealth to conduct a patient encounter was new for many health care providers. For there to be a successful transition to telehealth from traditional face-to-face visits with patients, there needs to be perceived value by health care providers. Prior to the pandemic, the uptake to utilize telehealth services was impacted by various barriers—funding, time, equipment, skills, and preferences of both patients and providers.^6^ There have been limited conducted studies related to the value of telehealth from the provider perspective; studies that have been conducted explored provider satisfaction with telehealth.^7^ Most providers perceive a value in telehealth as it relates to improving access to care, increasing convenience for patients, and the ability to provide care related to chronic disease management, medication refills, and mental health services.^6,7^ Telehealth can improve efficiency in the clinical arena with technology that allows for improved integration of home monitoring devices, improved connection between the patient and the provider, and guidelines to assist in the appropriate scheduling of patients.^8^ The ability to decrease provider burden will be dependent on how policies impacting reimbursement for telehealth services, asynchronous and synchronous, which give credit to providers for time spent reviewing reports, and calling patients to discuss results.^8,9^ More research needs to be done on telehealth’s impact on provider productivity and efficiency, reducing provider burnout, and improving work-life balance (reduction of after-hours work). Telehealth in the future needs to be leveraged to enhance the provider experience, with particular emphasis on determining optimum patient and visit types for telehealth encounters, the ideal balance between telehealth and in-person visits, and the administrative burden related to telehealth services.

### PATIENT EXPERIENCE

Patient satisfaction with provider and health care system is becoming recognized as an important component of value-based care. For many patients, the use of telehealth for a health care visit is new and can produce uncertainty regarding the accuracy of diagnosis. Many patients prefer in-person visits when there are concerns about a need for diagnostic accuracy, when there is higher acuity, and when specific examination is required.^4,10^ If patients perceive an increased cost or are older, they will be less likely to agree to a telehealth visit. If patients perceive there is not a need for an exam, feel their condition is minor or of low acuity, and feel there is little chance for diagnostic uncertainty, they are more likely to agree to a telehealth visit.^1,4,10^ In order to improve the patient experience related to telehealth, the patient’s choice for the type of visit needs to be considered. One potential means of addressing these preferences is for providers to offer alternating visits between telehealth and in-person. Providers, payers, and health systems can work together to assist patients in identifying the mode of health care delivery that will align with the patient’s choice and provide the most value.^3^

### QUALITY OF CARE

Quality of care is influenced by several factors, including patient satisfaction/experience, provider experience, and cost, which have been previously discussed. Health outcomes are also tied to quality of care, understanding the impact telehealth can potentially have on outcomes, and improving access to care and decreasing health inequities is important to understand. During the COVID-19 pandemic, telehealth was one of the main venues utilized for the delivery of care. Patients perceived telehealth-sustained access to care, and they perceived care quality and coordination to be maintained or improved.^9^ A systematic review conducted by Snoswell et al.^11^ found telehealth services can be equally or more clinically effective when compared with in-person care.

Access to telehealth was maintained for most individuals during the pandemic, but the expansion also highlighted health inequalities. Differences based on sociodemographics were identified. Non-White patients and those with comorbidities were found to have lower satisfaction with telehealth services when compared with White patients.^12^ Individuals who live in a community are deemed to have a high social vulnerability (such as poverty, lack of access to transportation, and crowded housing) and are less likely to have telehealth visits *via* video and may only have access using a telephone.^13^ Telehealth conducted with audio only has been shown to be inadequate in certain situations, for it may result in the delivery of subpar care.^13,14^ We currently do not know the true impact health inequalities have on outcomes and the use of telehealth. Telehealth can be a solution and is poised to address and improve health outcomes and have a positive impact on patients.

## Current Limitation and the Use of Emerging Technologies

The current telehealth system comes with limitations, particularly the inability to perform an actual physical exam. Health care providers currently need to rely on the patients’ ability to communicate effectively and report an accurate history of the problem. As telehealth evolves in the postpandemic era, the use of emerging technologies such as artificial intelligence (AI), wearable devices, and robotics can improve the value of care delivered *via* telehealth.^15–17^ These technologies also have the ability to improve outcomes, improve the quality of the telehealth experience for both patients and providers and move toward more patient-specific/personalized health care, and expand its use across the health care continuum.^17^

## Next Steps: Recommendations

To ensure telehealth is aligned with and becomes a core part of value-based care, models of care need to focus on improving outcomes, reducing costs, improving access and enhancing quality, and utilizing telehealth modalities:

(1) Invest in digital infrastructure. This infrastructure needs to expand broadband access and adopt advanced digital tools to support telehealth’s growth and ensure HIPAA compliance and patient privacy.
(2) Develop and promote national guidelines that standardize telemedicine implementation and reduce variability in adoption and ensure equitable access across systems.
(3) Enhance patient choice by allowing for patient flexibility to choose between telehealth and in-person visits. Patient education on choosing the most appropriate visit type will be an important component of this effort.
(4) Invest in research and the application of emerging technologies, that is, AI, related to improving the value of the telehealth experience for both providers and patients and quality outcomes.
(5) Implement targeted interventions like subsidized devices, improved digital literacy, and multilingual services that address health inequities and prevent telehealth from worsening current and/or creating new inequities.
(6) Focus on financial viability through advocating for transparent studies on telehealth’s cost-effectiveness and develop reimbursement policies that incentivize its long-term use.
(7) Expand research on provider experience and the impact telehealth has on provider productivity, burnout, and work-life balance to ensure sustainable integration into clinical practice.
(8) Strengthen long-term outcome research and invest in studies that evaluate telehealth’s long-term effects on chronic care management and its role in reducing health care disparities.
(9) Leverage EMR for population health *via* maximizing the EMR’s use of telemedicine to streamline patient management, improve chronic disease monitoring, and support population health efforts.

## Conclusions

Value-based care utilizing telehealth in the postpandemic era should be built on a framework that prioritizes health equity, patient choice, provider efficiency, and financial sustainability. By incorporating the components of emerging technologies such as AI, improved health outcomes, and the quality of care utilizing telehealth can be achieved through increased diagnostic accuracy and patient trust, as well as lower provider burden in the postpandemic era. The next steps and recommendations identified will help drive the ongoing evolution of telehealth in a way that maximizes its potential for improving care delivery, patient satisfaction, and overall health care value.
